# Factors affecting parental role adaptation in parents of preterm infants after discharge: a cross-sectional study

**DOI:** 10.3389/fpsyg.2024.1396042

**Published:** 2024-06-19

**Authors:** Jia Li, Xiaohong Zhang, Fei Ye, Xiaolin Cheng, Liping Yu

**Affiliations:** ^1^Department of Nursing, Zhongnan Hospital, Wuhan University, Wuhan, Hubei, China; ^2^School of Nursing, Center for Nurturing Care Research, Wuhan University, Wuhan, China; ^3^Department of Pediatrics, Xiangyang Central Hospital, Xiangyang, China; ^4^Department of Nursing, Fifth Affiliated Hospital, Southern Medical University, Guangzhou, China; ^5^Zhongnan Hospital, Wuhan University, Wuhan, Hubei, China

**Keywords:** parenting, preterm infant, parental role, adaptation, cross-sectional study

## Abstract

**Background:**

Parenting a preterm infant can be incredibly challenging and stressful, particularly in the first year after discharge. Desirable parental role adaptation leads to appropriate parenting behaviors and parent-infant interaction, which are essential to child health and development.

**Aim:**

To investigate the level of parental role adaptation and its influencing factors among parents of preterm infants in the first year after hospital discharge according to Belsky’s parenting process model among parents of preterm infants in the first year after hospital discharge.

**Methods:**

A cross-sectional study design was adopted using convenience sampling. Data were collected using the Parental Role Adaptation Scale (PRAS) in parents with preterm infants, the Perceived Social Support Scale (PSSS), the Coping Adaptation Processing Scale (CAPS-15), and a sociodemographic questionnaire. Descriptive statistics, non-parametric tests, Spearman correlation analyses, and multivariate linear regression were used to analyze the data.

**Results:**

In total, 300 Chinese parents were included in the analysis. In the multivariate analysis, first-time parent (*p* = 0.003), master’s degree and above (*p* = 0.042), coping adaptation processing (*p* = 0.000), residence location (towns: *p* = 0.019, city: *p* = 0.028), monthly family income (6000–10,000: *p* = 0.000, >10,000: *p* = 0.000), and perceived social support (*p* = 0.001) were all significant predictors of parental role adaptation and collectively accounted for 56.8% of the variation in parental role adaptation of parents with preterm infants (*F* = 16.473, *p* < 0.001). Coping adaptation processing mediated the relationship between perceived social support and parental role adaptation (95% bootstrap CI = 0.022, 0.130).

**Conclusion:**

Chinese parents of preterm infants experience a moderate level of parental role adaptation when their child is discharged from the hospital to home. Parents who are not first-time parents, have master’s degrees or above, live in towns or cities, have higher coping and adaptation abilities, have high monthly family income, and greater perceived social support have a higher level of parental role adaptation. Healthcare providers should pay more attention to parents with low socioeconomic status and encourage them to improve their coping and adaptation abilities and to utilize their formal and informal social support networks.

## 1 Introduction

Preterm babies are those who are born before 37 weeks of pregnancy (World Health Organization, 2024). In 2020, an estimated 13.4 million infants were delivered prematurely, and preterm birth rates vary from 4 to 16% of live births worldwide (Ohuma et al., 2023). In China, the preterm infant birth rate was 9.9% (Zhang et al., 2016). With the advancement of medical technology, neonatal mortality has been significantly reduced, and the survival rate and discharge rate of preterm infants have grown (Enlow et al., 2017). However, these newborns continue to encounter severe difficulties after discharge, and their follow-up and family care are critical (Boykova, 2016b).

Parents are naturally responsible for making decisions about the daily care and parenting of their children. According to the parenting process model developed by Belsky (1984) and Taraban and Shaw (2018), parenting is multi-determined and impacted by the interaction of three distinct domains: parental characteristics (e.g., personality, education level, and psychological well-being), social contextual factors (e.g., marital relationship quality, employment status, and social support) and child characteristics (e.g., temperament, age, and sex). This theoretical model provides a relatively comprehensive insight to understand the influencing factors of parenting. Identifying factors that influence adaptation to parenthood in parents of preterm infants after discharge can improve follow-up care and expand understanding of how parents cope and adapt to caring for a preterm infant.

Child’s health and development outcomes were influenced by both biological factors (gestational age, illness, and fragility) and environmental factors (family care, parenting, and parental psychological traits) (Aboud and Yousafzai, 2015). Parenting is one of the most important environmental predictors of the child’s quality of life and development (Shah et al., 2013; Treyvaud et al., 2016). A systematic review showed that parental factors also influence neurodevelopmental outcomes of preterm infants (Neel et al., 2018). Raising a preterm infant is a demanding endeavor, and parents confront numerous problems when their preterm infants are discharged from the hospital (Boykova, 2016a). In addition to daily needs such as feeding and cleaning, parents of preterm infants must satisfy higher levels of care needs on their own, including medical needs such as drug management, use of home medical equipment and instruments, and regular follow-up (Murdoch and Franck, 2012; White et al., 2017). Furthermore, because most neonatal intensive care units (NICUs) in China implement closed management, parents and infants are separated for an extended period after birth, which delays parental role acquisition and makes it harder to establish parent–child interactions (Phillips-Pula et al., 2013). These issues may disrupt the usual process of parental role acquisition (Whittingham et al., 2014), leading to parents struggling with role adaptation, barriers to the formation of a parent-infant relationship, and a perception of insufficient caregiving capacities (Premji et al., 2017; Bright et al., 2020).

Parental role adaptation refers to the conceptualization and establishment of parental roles, which is a process of developing new identities and parenting care behaviors and integrating parental roles into pre-existing role models (Mercer, 2004; Barimani et al., 2017). Lack of parental role adaptation can reduce parents’ abilities to care for their infants, undermine their parental responsibilities, and result in decreased parent–child attachment (Barr, 2008; Solmeyer and Feinberg, 2011). Undesirable parental role transitions can also impair children’s health and development (Rieves et al., 2016). However, successful parental role adaptation fosters parents’ self-confidence and satisfaction (Emmanuel et al., 2008), which are crucial to the quality of parenting behavior and have an important impact on the health, psychological, and social development of children (Barimani et al., 2017; Heydarpour et al., 2017; Rafii et al., 2020). Therefore, paying attention to the parental role adaptation of parents with preterm infants is critical for enhancing the developmental outcomes of preterm infants.

Existing studies have primarily focused on parents’ negative psychological and emotional experiences while ignoring parents’ positive opinions on adapting and coping in this challenging situation (Adama et al., 2016; Boykova, 2016a). Previous studies, which largely concentrated on the experiences of parents of premature infants during hospitalization (Loewenstein et al., 2019). There is insufficient attention paid to the period after preterm infants are discharged from the hospital and returned home. In addition, existing research on parental role adaptation generally focused on full-term infants and explored the unilateral maternal role adaptation (Wu and Lu, 2009; Javadifar et al., 2013). There is a lack of attention to the vulnerable group of parents of premature infants. Furthermore, available research was predominantly conducted in Western countries (Boykova, 2016a), with little specific attention paid to the same issues in the context of Chinese culture. The parenting process model developed by Belsky (1984) emphasizes that parenting is multiply determined and is influenced by the characteristics of the parent, child, and social context, which is an appropriate tool for explaining the process by which parenting occurs and its determinants. Given that parental role adaptation may be associated with each of the above factors, examining parental role adaptation together with these related factors adds a layer of comprehensiveness. Thus, the first purpose of this study is to understand the level of parental role adaptation of premature infants after discharge from the hospital. The second is to identify its influencing factor by using Belsky’s parenting process model.

## 2 Materials and methods

A cross-sectional study design was adopted, and the Strengthening the Reporting of Observational Studies in Epidemiology (STROBE) guidelines were followed in reporting the study (Supplementary File S1) (von Elm et al., 2007).

### 2.1 Participants

This study was conducted between January 21, 2022, and August 30, 2022, in Wuhan (the capital of Hubei Province in central China) and Xiangyang (a city in Hubei Province) using convenience sampling. The participants were recruited from the preterm follow-up clinic of two university teaching hospitals that each provide regular services for preterm infants discharged from the NICU. Parents receive monthly alerts from the hospital asking them to bring their children for medical check-ups.

The study population consisted of parents of preterm infants who were discharged from the NICU within the past year. The first year following discharge from hospital to home is a critical catch-up phase for the health and development of preterm infants (Ng et al., 2019), and the most frequent period of adverse events after discharge, including readmission and emergency/outpatient use (Mourani et al., 2014). In addition, it is a critical period for the development of parental roles. The following were the inclusion criteria for parents: having preterm infants with gestational age less than 37 weeks; being at least 18 years old; and being able to read and complete the questionnaires. The following were the exclusion criteria: having infants with congenital malformations, genetic diseases, or other serious complications; not having infants who were admitted to the NICU; and having a history of mental illness.

The sample size was estimated using the formula *N* = Z^2^_1-α/2_ × σ^2^/δ^2^ (Johnston et al., 2019) assuming a type I error (α) of 5%. The standard deviation (σ) and precision level (δ) were calculated to be 13.06 and 1.71, respectively, based on a previous investigation (Huang et al., 2023). Therefore, the minimum sample size needed was 225 (1.96^2^ × 13.06^2^/1.71^2^), which was increased to 270 accounting for a potential 20% attrition rate.

### 2.2 Data collection procedure

Two qualified researchers (J.L and XH.Z) collected the data at two preterm infant follow-up clinics in Wuhan and Xiangyang, respectively. To ensure the data’s authenticity and quality and to eliminate participants’ understanding bias, a pilot study was conducted with 15 participants to evaluate their comprehension of the questionnaires prior to formal data collection. Some minor modifications were made following the pilot including the instructions for the questionnaire and some ambiguous statements. When parents visited the preterm infant’s follow-up clinics for routine physical examinations and health consultations, the researchers recruited the participants who met the inclusion criteria and explained the purpose, content, and significance of the research. After parents provided their written informed consent, they were given a questionnaire to complete. While filling out the questionnaire, a researcher swiftly answered any queries the participants had and after completing the questionnaire, the researcher returned to the participants to ensure that all the items had been completed. If any were missing, the participants were prompted to complete them. The questionnaire was required to be completed within approximately 20–25 min after receipt.

### 2.3 Instruments

#### 2.3.1 The sociodemographic questionnaire

The self-designed sociodemographic questionnaire used comprised two components. The first portion covered parental characteristics including gender, age, education level, and employment status, and the second portion covered preterm infants’ characteristics including gender, type of delivery, gestational age, and birthweight.

#### 2.3.2 The parental role adaptation scale (PRAS) in parents with preterm infants

The PRAS in parents in preterm infants was developed by Li et al. (2023) and was used to evaluate parents’ self-reported degree of adaptability to parental role responsibilities after their preterm infants were discharged from the NICU. It includes three aspects: role perception, role performance, and balance between diverse social roles. The scale has 28 items over 7 dimensions: role perception, role performance (health care, nutrition care, safety care, responsive care, and early learning care), and role balance. The parents rated their responses on a 5-point Likert scale, ranging from 1 (strongly disagree) to 5 (strongly agree); the total score can therefore range from 28 to 140, with higher scores indicating a better perception of parental role adaptation. The PRAS scale has good psychometric properties, with a Cronbach’s *α* coefficient of 0.935 and a half reliability of 0.822. Its Cronbach’s *α* coefficient for the current study was 0.937.

#### 2.3.3 The perceived social support scale (PSSS)

The PSSS was developed by Blumenthal et al. (1987) and translated into Chinese version by Zhang et al. (2018). It was developed to assess an individual’s perceived multi-level social support. The scale contains 3 dimensions: family support, friend support, and other support, with a total of 12 items scored using a 7-point Likert scale (1–7 points) where the overall score indicates the degree of perceived social support. Three levels of the PSSS score are available for assessment: low (score 12–36), medium (score 37–60), and high (score 62–84). The Cronbach’s *α* coefficient of the Chinese version of the PSSS is 0.840 (Zhang et al., 2018) and for this study was 0.905.

#### 2.3.4 The coping adaptation processing scale (CAPS-15)

The 15-item Coping and Adaptation Processing Scale (CAPS-15) was simplified by Roy et al. (2016) based on the original Coping and Adaption Processing Scale (47-item). It is a useful instrument for measuring coping and adaptability when people dealing with difficulties or crises. This study used the Chinese version of the Coping Adaptation Processing Scale (CAPS-15) that was translated and revised by Li et al. (2018). The scale has one dimension and 15 items are scored using a 4-point Likert scale. Individuals evaluate how frequently they employ relevant coping strategies when confronted with difficulties or crises as never, rarely, sometimes, and often, with scores ranging from 1 to 4 respectively, for a total possible score of 15 to 60 points. The higher the score, the better the capacity to cope with and adapt to the situation. The Cronbach’s *α* coefficient of the scale is 0.815, and in the current was 0.788.

### 2.4 Data analysis

Data were analyzed using the IBM SPSS Statistics 27.0 (IBM Corp, Armonk, NY, United States) and Process Macro version 3.4.1. Descriptive statistics were presented as frequencies and scores. Frequencies and percentages were used to describe categorical variables of parental and preterm infants’ characteristics, while median value and interquartile Range (IQR) were used to describe the non-normally distributed continuous scale scores. In the univariate analysis, non-parametric tests were used to compare PRAS scores and socio-demographic characteristics, while Spearman correlation analysis was employed to test the associations between PSSS, CAPS, and PRAS. Hierarchical regression model analysis was performed to explore factors that were independently associated with the PRAS. The mediation effect of independent variables and PRAS was examined by the Hayes’ Process Tool (Hayes, 2018). To determine the significance of the mediation effect, a 95% bootstrap confidence interval (CI) with 5,000 bootstrapped samples was used. The threshold for statistical significance was set at *p* < 0.05 for all statistical tests.

### 2.5 Ethical considerations

The Wuhan University Medical Ethics Committee (NO. 2020 YF 0062) approved this work. Parents of preterm infants signed the informed consent form before they completed the questionnaires, and each participant had the option to continue or withdraw from the study at any time. All information collected from the participants was kept anonymous and held confidentially.

## 3 Results

### 3.1 Sociodemographic characteristics of the participants

During the recruitment period, 346 eligible parents were contacted, 32 of whom declined to participate in the survey. Then during the data analysis period, 14 participants were excluded due to incomplete questionnaires. Finally, a total of 300 parents were included. Most participants were mothers (73%) and first-time parents (62.3%). Additionally, 50.3% of parents were aged ≤30 years and 45.0% of parents were aged 31–40 years. Approximately half of parents had a master’s degree and above (49.4%), and the proportions of parents who rated their satisfaction with the discharge service and follow-up service as good were 73.7 and 69.3%. The sociodemographic characteristics of the parents and infant characteristics are shown in Table 1.

**Table 1:** Sociodemographic characteristics of the participants (*n* = 300).

Variables	*n* (%)
Gender	
Male	81 (27.0)
Female	219 (73.0)
Age (years)	
≤30	151 (50.3)
31–40	135 (45.0)
≥41	14 (4.7)
First time parent	
No	113 (37.7)
Yes	187 (62.3)
Education level	
Primary education	10 (3.3)
Secondary education	52 (17.4)
Bachelor’s degree	98 (32.6)
Master’s degree and above	140 (46.7)
Employment status	
Unemployed	103 (34.3)
Part-time job	32 (10.7)
Full-time job	165 (55.0)
Marital status	
Married	295 (98.3)
Single/divorced/widowed	5 (1.7)
Residence location	
Rural areas	43 (14.3)
Towns	108 (36.0)
Cities	149 (49.7)
Monthly family income (CNY)	
<3,000	23 (7.6)
3,000–6,000	71 (23.6)
6,000–10,000	115 (38.4)
>10,000	91 (30.4)
Living with the grandparents	
Yes	102 (34.0)
No	198 (66.0)

### 3.2 The relationship between parental role adaptation and demographic characteristics

According to the univariate analysis (Table 2), parental role adaptation was associated with parenting experience, education level, employment status, residence location, monthly family income, satisfaction with discharge and follow-up service, and children’s gestational age, birthweight in grams, and readmission times. In univariable analyses, first-time parents (*Z* = −2.639, *p* = 0.008), a lower education level (*H* = 31.973, *p* = 0.000), parents with a part-time job (*H* = 15.621, *p* = 0.000), parents who lived in rural (*H* = 46.391, *p* = 0.000), lower monthly family income (*H* = 125.375, *p* = 0.000), dissatisfied with the discharge service (*Z* = −5.377, *p* = 0.000), dissatisfied with the follow-up service (*Z* = −5.377, *p* = 0.000) and children with lower gestational age (*H* = 19.066, *p* = 0.000), lower birthweight (*H* = 27.044, *p* = 0.000) and more readmission times (*H* = 24.837, *p* = 0.000) were significantly associated with a lower level of parental role adaptation.

**Table 2:** Correlations between parental role adaptation and demographic characteristics (*n* = 300).

Variables	*n* (%)	Median (IQR)	*Z*/*H*	*p-*value
Parental characteristics				
Gender				
Male	81 (27.0)	117.00 (15.00)	−0.565	0.572
Female	219 (73.0)	116.00 (20.00)		
Age (years)				
≤30	151 (50.3)	118.00 (19.00)	2.435	0.296
31–40	135 (45.0)	114.00 (17.00)		
≥41	14 (4.7)	120.00 (16.75)		
First time parent				
No	113 (37.7)	118.00 (21.00)	−2.639	0.008^**^
Yes	187 (62.3)	115.00 (19.00)		
Education level				
Primary education	10 (3.3)	105.00 (11.50)	31.973	0.000^***^
Secondary education	52 (17.4)	110.50 (19.50)		
Bachelor’s degree	98 (32.6)	111.50 (19.25)		
Master’s degree and above	140 (46.7)	120.00 (14.00)		
Employment status				
Unemployed	103 (34.3)	113.00 (18.00)	15.621	0.000^***^
Part-time job	32 (10.7)	110.00 (25.00)		
Full-time job	165 (55.0)	119.00 (18.00)		
Marital status				
Married	295 (98.3)	116.00 (18.00)	−0.702	0.483
Single/divorced/widowed	5 (1.7)	106.00 (33.00)		
Residence location				
Rural	43 (14.3)	105.00 (10.00)	46.391	0.000^***^
Town	108 (36.0)	116.00 (17.75)		
City	149 (49.7)	120.00 (17.00)		
Monthly family income (CNY)				
<3,000	23 (7.6)	111.00 (11.08)	125.375	0.000^***^
3,000–6,000	71 (23.6)	114.00 (11.25)		
6,000–10,000	115 (38.4)	118.00 (13.02)		
>10,000	91 (30.4)	124.50 (11.25)		
Living with the grandparents				
Yes	102 (34.0)	114.00 (19.00)	−1.297	0.195
No	198 (66.0)	118.00 (17.25)		
Satisfaction with the discharge service				
Satisfied	85 (28.3)	110.00 (11.50)	−7.041	0.000^***^
Dissatisfied	215 (71.7)	120.00 (18.00)		
Satisfaction with the follow-up service				
Satisfied	99 (33.0)	111.00 (15.00)	−5.377	0.000^***^
Dissatisfied	201 (67.0)	120.00 (19.00)		
Preterm infants’ characteristics				
Gender				
Female	112 (37.3)	117.50 (18.00)	0.822	0.936
Male	136 (45.4)	115.50 (18.00)		
Twins or Multiples-male	22 (7.3)	117.50 (20.25)		
Twins or Multiples -female	17 (5.7)	122.00 (13.50)		
Pigeon pair	13 (4.3)	112.00 (18.50)		
Type of delivery				
Normal vaginal delivery (NVD)	102 (34.0)	117.00 (21.00)	−0.089	0.929
Cesarean section	198 (66.0)	116.00 (16.25)		
Gestational age (weeks)				
Extremely preterm (less than 28 weeks)	17 (5.7)	108.00 (15.00)	19.066	0.000^***^
Very preterm (28 to less than 32 weeks)	87 (29.0)	113.00 (19.00)		
Moderate to late preterm (32–37 weeks)	196 (65.3)	119.00 (17.00)		
Multiple birth				
Yes	56 (18.7)	117.50 (16.75)	−0.267	0.790
No	244 (81.3)	116.00 (18.00)		
Birth weight in grams				
<1,000	15 (5.0)	105.00 (15.00)	27.044	0.000^***^
1,000–1,500	63 (21.0)	112.00 (15.00)		
1,500–2,500	158 (52.7)	117.50 (19.00)		
≥2,500	64 (21.3)	120.00 (17.75)		
Length of hospital stay (days)				
<7 days	37 (12.3)	121.00 (17.00)	3.382	0.184
7–14 days	80 (26.7)	115.50 (16.00)		
≥14 days	183 (61.0)	116.00 (20.00)		
Readmission times				
0 times	210 (70.0)	119.00 (18.00)	24.837	0.000^***^
1–2 times	79 (26.3)	113.00 (20.00)		
≥3 times	11 (3.7)	105.00 (13.00)		
Infant feeding pattern				
Exclusive breastfeeding	70 (23.3)	118.00 (16.25)	4.181	0.243
Breastfeeding plus breast milk booster	20 (6.7)	123.00 (17.50)		
Partial breastfeeding	87 (29.0)	114.00 (20.00)		
Bottle feeding	123 (41.0)	118.00 (17.00)		

*Z*/*H*, Mann–Whitney *Z* test/Kruskal–Wallis *H* test; ^**^p < 0.01, ^***^p < 0.001.

### 3.3 Association between parental role adaptation, perceived social support, and coping adaptation processing

The median (IQR) scores of parental role adaptation, perceived social support, and coping adaptation processing were 116.00 (18.00), 72.00 (15.00), and 46.00 (8.00), respectively. Each dimension’s median score of the study variables is presented in Table 3. In Table 4, Spearman’s correlation analysis shows that the PRAS score was positively significantly correlated with both perceived social support (*r* = 0.420, *p* < 0.01) and Coping Adaptation Processing (*r* = 0.412, *p* < 0.01). This finding implies that the higher the parent’s level of perceived social support, the higher their level of PRA, as well as the higher the level of coping and adaptation ability, the higher the parent’s degree of PRA.

**Table 3:** Scores on the parental role adaptation, perceived social support, and coping adaptation processing scales (*n* = 300).

Variables	Medium (IQR)	Range (min, max)
PRA total score	116.00 (18.00)	(77.00, 140.00)
Role perception	17.00 (3.00)	(11.00, 20.00)
Role performance-health	12.00 (3.00)	(6.00, 15.00)
Role performance-nutrition	17.00 (3.00)	(11.00, 20.00)
Role performance-safety	16.00 (3.00)	(8.00, 20.00)
Role performance-responsive caring	21.00 (4.00)	(12.00, 25.00)
Role performance-early learning	17.00 (3.75)	(8.00, 20.00)
Role balance	16.00 (5.00)	(5.00, 20.00)
PSSS total score	72.00 (15.00)	(27.00, 154.00)
Family support	24.00 (5.00)	(8.00, 85.00)
Friend support	24.00 (6.00)	(6.00, 98.00)
Other support	24.00 (4.75)	(11.00, 59.00)
CAPS-15 total score	46.00 (8.00)	(32.00, 75.00)

PRA, parental role adaptation scale in parents with preterm infants; PSSS, perceived social support scale; CAPS-15, coping adaptation processing scale; IQR, interquartile range.

**Table 4:** Correlations between PRA and primary variables (*n* = 300, *r*).

Variables	PSSS total score	Family support	Friend support	Other support	CAPS-15 total score
PRA total score	0.420^***^	0.406^***^	0.407^***^	0.393^***^	0.412^***^
Role perception	0.313^***^	0.286^***^	0.297^***^	0.293^***^	0.269^***^
Role performance-health	0.230^***^	0.219^***^	0.218^***^	0.210^***^	0.284^***^
Role performance-nutrition	0.344^***^	0.318^***^	0.333^***^	0.333^***^	0.357^***^
Role performance-safety	0.217^***^	0.230^***^	0.170^***^	0.251^***^	0.390^***^
Role performance-responsive caring	0.271^***^	0.290^***^	0.249^***^	0.264^***^	0.351^***^
Role performance-early learning	0.375^***^	0.357^***^	0.380^***^	0.344^***^	0.218^***^
Role balance	0.414^***^	0.381^***^	0.420^***^	0.357^***^	0.331^***^

PRA, parental role adaptation scale in parents with preterm infants; PSSS, perceived social support scale; CAPS-15, coping adaptation processing scale, ^***^p < 0.001.

### 3.4 Influencing factors for parental role adaptation

Hierarchical regression analysis was employed to explore the independent influencing factors for parental role adaptation (Table 5). Regression models were constructed using statistically significant factors from the univariate analysis as independent variables and parental role adaptation as the dependent variable according to Belsky’s parenting process model. Regression equation 1 considers the personal characteristics of the parents (first-time parent, educational level, employment status). The results show that 34.2% of the variance in parental role adaptation could be explained by the characteristics of the parents. In equation 2, the socio-contextual factors (residence location, monthly family income, satisfaction with the discharge service, satisfaction with the follow-up service, and perceived social support) were involved, and the results show that they explained 20% of the variation in parental role adaptation. Equation 3 included infant characteristics (gestational age, birth weight in grams, readmission times), and these results demonstrate that they explained 2.6% of the variance of parental role adaptation. In the final model, first-time parent (*β* = −3.272, *p* = 0.003), education level (master’s degree and above: *β* = 6.187, *p* = 0.042), coping adaptation processing (*β* = 0.395, *p* = 0.000), residence location (towns: *β* = 3.990, *p* = 0.019, city: *β* = 3.919, *p* = 0.028), monthly family income (6000–10,000: *β* = 9.038, *p* = 0.000, >10,000: *β* = 14.862, *p* = 0.000), and perceived social support (*β* = 0.151, *p* = 0.001) were significant predictors of parental role adaptation, which collectively accounted for 56.8% of the variation in parental role adaptation of parents with preterm infants (*F* = 16.473, *p* < 0.001).

**Table 5:** Multiple linear regression analysis of parental role adaptation (*n* = 300).

Variable		Model 1				Model 2				Model 3			
		Unstandardized coefficients		*t*	*p-*value	Unstandardized coefficients		*t*	*p-*value	Unstandardized coefficients		*t*	*p-*value
		*B*	*SE*			*B*	*SE*			*B*	*SE*		
First time parent	No	Ref				Ref				Ref			
	Yes	−5.364	1.222	−4.39	0.000	−3.768	1.052	−3.581	0.000	−3.272	1.079	−3.033	0.003
Education level	Primary education	Ref				Ref				Ref			
	Secondary education	7.194	3.401	2.115	0.035	3.161	2.954	1.07	0.285	2.905	2.955	0.983	0.326
	Bachelor’s degree	11.566	3.277	3.53	0.000	5.155	2.898	1.779	0.076	5.34	2.923	1.827	0.069
	Master’s degree and above	17.249	3.28	5.259	0.000	6.64	3.011	2.205	0.028	6.187	3.033	2.04	0.042
Employment status	Unemployed	Ref				Ref				Ref			
	Part-time job	−5.372	2.062	−2.606	0.010	−3.991	1.775	−2.248	0.025	−3.055	1.790	−1.707	0.089
	Full-time job	−0.629	1.389	−0.453	0.651	−1.098	1.206	−0.910	0.364	−0.939	1.204	−0.780	0.436
CAPS-15		0.722	0.105	6.864	0.000	0.417	0.095	4.398	0.000	0.395	0.094	4.190	0.000
Residence location	Rural					Ref				Ref			
	Town					4.070	1.689	2.410	0.017	3.990	1.695	2.354	0.019
	City					4.121	1.758	2.344	0.020	3.919	1.779	2.203	0.028
Monthly family income (CNY)	<3,000					Ref				Ref			
	3,000–6,000					3.188	2.211	1.441	0.151	2.598	2.251	1.154	0.250
	6,000–10,000					9.805	2.240	4.378	0.000	9.038	2.259	4.000	0.000
	>10,000					15.542	2.382	6.525	0.000	14.862	2.413	6.160	0.000
Satisfaction with the discharge service	1					Ref				Ref			
	2					−0.569	1.637	−0.348	0.728	−0.224	1.629	−0.138	0.891
Satisfaction with the follow-up service	1					Ref				Ref			
	2					2.109	1.539	1.370	0.172	2.048	1.530	1.338	0.182
PSSS						0.157	0.044	3.599	0.000	0.151	0.044	3.427	0.001
Gestational age (weeks)	extremely preterm (less than 28 weeks)									Ref			
	very preterm (28 to less than 32 weeks)									2.407	2.721	0.884	0.377
	moderate to late preterm (32 to 37 weeks)									0.392	2.917	0.134	0.893
Birth weight in grams	<1,000									Ref			
	1,000–1,500									−2.555	2.879	−0.887	0.376
	1,500–2,500									2.113	3.021	0.699	0.485
	≥ 2,500									3.144	3.242	0.970	0.333
Readmission times	0 times									Ref			
	1–2 times									−0.307	1.201	−0.256	0.798
	≥3 times									−3.191	2.774	−1.150	0.251
*R* ^2^		0.342				0.542				0.568			
Adjust *R*^2^		0.326				0.518				0.533			
*F*		21.604^***^				22.316^***^				16.473^***^			

PSSS, perceived social support scale; CAPS-15, coping adaptation processing scale, ^***^p < 0.001.

**Figure 1:**
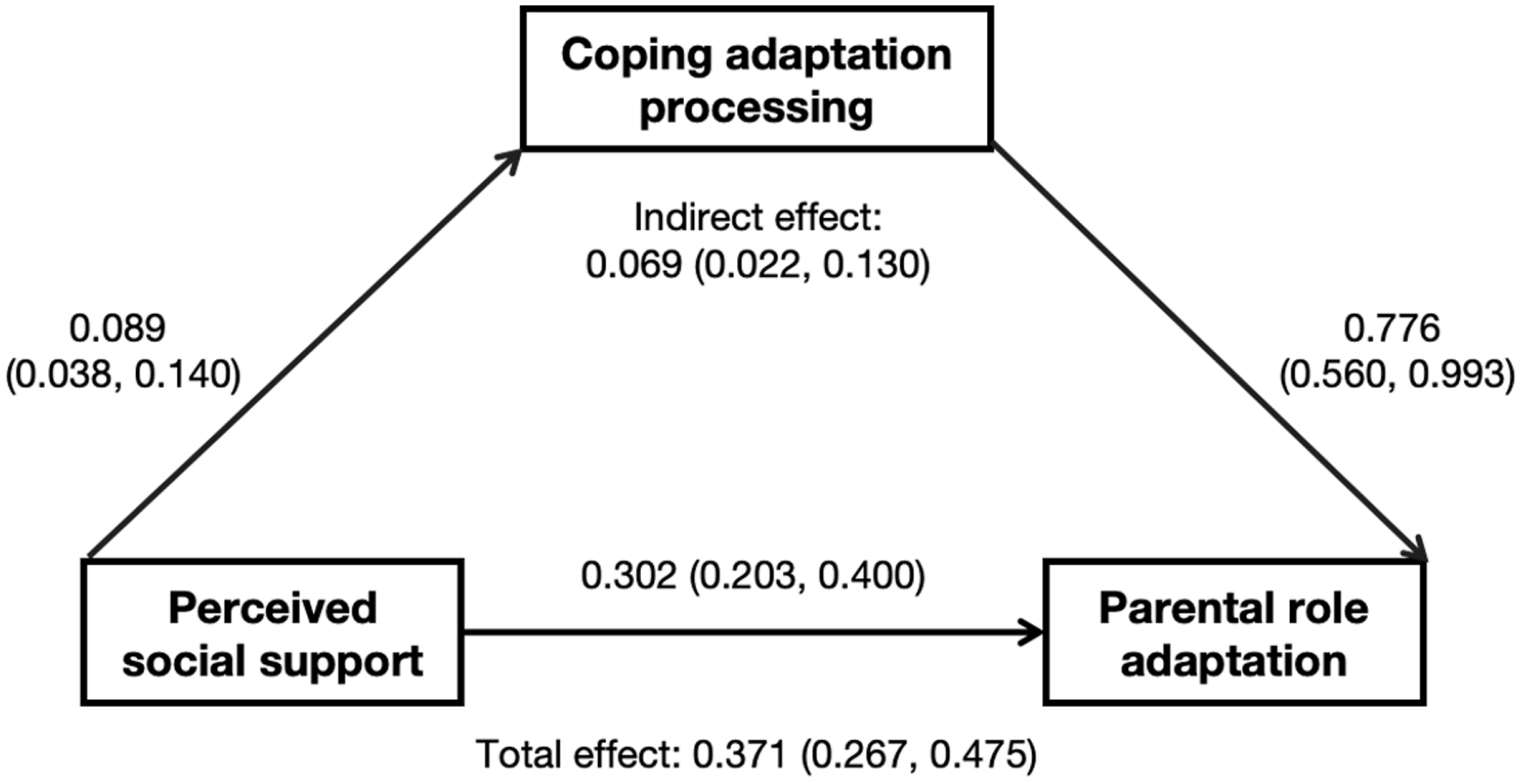
Direct and indirect effect of parental characteristics and socio-contextual factors on parental role adaptation in parents with preterm infants.

Based on the findings of the regression analysis, a mediation analysis was conducted between perceived social support (PSSS), coping adaptation processing (CAPS), and parental role adaptation (PRAS). Figure 1 shows the results of the bootstrapping method which confirmed the significance of the indirect effects of PSSS on PRAS through CAPS (95% bootstrap CI = 0.022, 0.130). The mediating effects were statistically significant since the 95% bootstrap confidence intervals did not include zero between their lower and upper limits.

## 4 Discussion

The aim of this study was to investigate the levels of parental role adaptation in parents of preterm infants, as well as to examine the factors affecting parental role adaptation using Belsky’s parenting process model. Our findings indicate that there is a moderate degree of parental role adaptation among parents of preterm infants. According to Belsky’s parenting process model which states that parenting is determined by the interaction of three distinct domains, we also found that first-time parents, education level, coping adaptation processing, residence location, monthly family income, and perceived social support are influencing factors of parental role adaptation. However, we only discovered that parental and social contextual domains were affecting variables, child characteristics were not recognized as an influencing element in parenting. Nonetheless, the findings are clinically important as little is known about parental role adaptation among parents with preterm infants in the first year after hospital discharge. Although our findings are consistent with those of previous studies (Heydarpour et al., 2017; Shani-Sherman et al., 2019), the time period used for this study is unique.

To the best of our knowledge, our study is the first to investigate the level of parental role adaptation among parents of preterm infants in China within the first year after discharge. Parents of preterm infants demonstrated a moderate level of parental role adaptation in this study. This finding is in line with Huang et al. who found that parents of premature infants had a moderate parenting sense of competence (Huang et al., 2023). In addition, some previous qualitative studies have also found that parents of preterm infants have difficulties in their role adaptation (Murdoch and Franck, 2012; White et al., 2017; Neel et al., 2018). The underlying mechanism might be that parenting a preterm infant could be extremely demanding and difficult for parents, particularly in the first year following discharge (Spielman and Taubman-Ben-Ari, 2009). Parents could view preterm birth and the hospitalization of their infants as a crisis since it disrupted their pregnancy unexpectedly and forced them to become parents too soon (Boykova, 2016b). In China, parents are not allowed to visit the NICU. Thus, decreasing parental role adaptation can result from the separation of child and parent and the resultant lack of parent-infant interaction, which makes it difficult for parents to fulfill their parental responsibilities at home later (Schmöker et al., 2020). Our study also provides the groundwork for future research into the parenting of preterm infants. The instrument used in this study was a population-specific scale that was administered for the first time to parents of preterm newborns. Further studies are required to investigate parental role adaptation in parents of premature infants across cultures and social contexts. Furthermore, universal screening for parental role adaptation among parents of preterm infants should be included in routine care prior to discharge, and the results of this initial screening should dictate future screening and follow-up care.

In this study, we found that being a first-time parent was an influencing factor for parental role adaptation among parents with preterm infants. Specifically, first-time parents had a lower level of parental role adaptation than existing parents (parents with parenting experience). Previous research has also demonstrated that existing parents have better discharge readiness (Gong et al., 2021; Sun et al., 2024) and higher coping abilities after discharge (Luo, 2022) than first-time parents, which is consistent with our findings. The reason is that existing parents are more familiar with all elements of parenting (health, nutrition, safety, responsive care, and early learning opportunities) and are better positioned to deal with emergencies after premature infants are discharged from the hospital (Nunes-Costa et al., 2020). Conversely, first-time parents struggle to meet the daily care and disease care demands of premature newborns due to a lack of parenting expertise (McLeish et al., 2020). Therefore, health professionals should pay more attention to first-time parents of preterm infants and provide more help to them. The role adaptation level of novice parents of premature infants should be more comprehensively assessed in future research, but in the meantime, clinicians should also help them become more confident by providing parenting manuals, training, and online parenting consultations. This may help them adjust to their parental roles more successfully and ensure the preterm infants’ development and well-being post-discharge.

Education level was also found to be a significant influencing factor in parental role adaptation of preterm infants. Parents with better education backgrounds, in contrast to those with lower education levels, exhibited greater parental role adaptation. This finding has some similarities to previous studies (Shrooti et al., 2016). One possible explanation is that parents with higher education levels can more actively acquire relevant parenting knowledge to care for their preterm infants, perceive more social support, and make full use of resources than lower-educated parents, resulting in greater confidence in their parenting roles (Cutrona and Troutman, 1986; Teti and Gelfand, 1991). On the contrary, parents with lower educational levels have limited access to parenting-related knowledge and are easily overwhelmed when confronted with the various conditions of premature infants, resulting in poor role adaptation (Seo, 2006). Healthcare providers should thus pay more attention to parents with lower levels of education and provide them with more parenting-related counsel and education to improve their parental role adaption.

Positive associations were found between coping adaptation processing and parental role adaptation. This finding may be explained by the fact that internal psychological resources can reduce suffering in people dealing with severe medical events (Shani-Sherman et al., 2019). Coping adaptation processing refers to an individual’s generalized coping and adaptation ability when facing difficulties and crises (Roy et al., 2016). When the general coping and adaptation level of parents of preterm infants is high, it means that they are more confident in their parenting abilities and can effectively handle emergencies. When it is low, they have insufficient confidence and ability to care for their preterm infants, resulting in a low level of parental role adaptation (Wu et al., 2016; Liyana Amin et al., 2018; Banik et al., 2021). This is a novel finding, but it is consistent with earlier studies that have reported an association between parental psychosocial characteristics with parenting ability. For example, Barkin et al. (2019) found that lower Edinburgh Postnatal Depression Scale scores were significantly correlated with increased levels of maternal functioning. The Heydarpour et al. (2017) study’s findings also revealed that self-efficacy is one of the most important factors that influence adaptation to the maternal role, and Pan et al. (2022) found that the resilience level of parents of preterm infants was an influencing factor on parents’ coping ability after their preterm infants’ discharge. In the present study, we found that the higher the general coping and adaptation level of parents of preterm infants, the higher their parental role adaptation level. Further qualitative and qualitative research is needed to explore any other positive psychological resources that can influence parenting among parents of preterm infants in the first year after discharge.

The present study showed that parents who lived in towns/cities had higher parental role adaptation scores than those who lived in rural. This is a novel finding, as no previous research has even investigated this relationship. A potential rationale for this could be that Chinese medical resources differ significantly between urban and rural areas. Parents of preterm infants who live in more urban areas have more convenient medical and parenting consultation resources. According to data from the two hospitals where this study was conducted, two-thirds of the patients in their Preterm Infant Follow-up Center were from Wuhan and Xiangyang city, and only a small number were from rural areas. This demonstrates that compared to parents of preterm infants living in rural areas, most urban parents could be followed up with regularly and obtain more easily valuable professional knowledge and guidance on parenting preterm infants, which led them to have a higher level of parental role adaptation. The results of this study also showed that parents with greater monthly family income had higher levels of parental role adaptation, which is in line with previous studies. For example, Delgado Galeano and Villamizar Carvajal (2016) found a positive association between income range and level of coping in mothers of preterm infants following hospital discharge, and Rajabi et al. (2018) discovered that limited family income reduced adaptation to difficulty and dissatisfaction in mothers with late-preterm infants. Holden et al. (2018) also found that higher economic status increases the self-efficacy of mothers with preterm infants. This finding could be due to the fact that parents of preterm infants with higher family incomes are more likely to have access to a variety of medical, social media, and information resources, allowing them to continually accumulate care experience and adapt to their parental roles.

A significant positive relationship was also found between perceived social support and parental role adaption, which is again consistent with previous research. It is commonly known that great parenting behaviors and parent well-being are correlated with higher levels of social support (Angley et al., 2015). Huang et al. (2023) found that social support is significantly correlated with parenting sense of competence in parents with preterm infants. Similarly, a meta-synthesis of qualitative studies revealed that social support following discharge improved parents’ confidence and capacity for care (Adama et al., 2016). Formal social support structures can provide both reassurance and professional parenting advice, as well as guidance on child-rearing to parents with preterm infants, increasing caring confidence and helping with the transition to parenthood (Ncube et al., 2016; Heydarpour et al., 2017). Furthermore, informal social support from friends, family, and significant others can also give parents of preterm infants the necessary physical, emotional, and informational support, allowing them to spend more time with their babies and assisting their parental role adaptation (Murdoch and Franck, 2012; Shani-Sherman et al., 2019). Parents who feel supported may also be less stressed out about parenting and as a result have greater confidence in their parental roles (Angley et al., 2015). Therefore, the findings of this study emphasize the necessity for increased awareness among healthcare professionals regarding the importance of considering and incorporating parents’ larger social support networks into the care they provide since parents rely on these networks during the first year following hospital discharge.

In addition to the above results, we found a significant indirect relationship via coping adaptation processing between perceived social support and parental role adaptation, indicating that their association was partly mediated by coping adaptation processing. These findings indicate that when parents perceive higher levels of support from their social network, they may be more confident in dealing with difficulties and crises, which may in turn lead to greater adaptability to accepting their parental roles and performing parenting behaviors when their preterm infants are discharged home. Perceived social support can influence the parental role adaptation of parents of premature infants by influencing their general coping and adaptation skills as well, which is in accordance with Belsky’s parenting process model in which the social contextual factors can either directly affect parenting or indirectly affect parenting by influencing individual psychological characteristics (Belsky, 1984). The benefits of social network contact on parental functioning are mediated by the parent’s psychological well-being. In line with this theory, Taraban and Shaw (2018) argued that the support provided by social networks can enhance self-esteem and as a result increase the patience and sensitivity that parents exercise in the parenting roles. A variety of contextual factors influenced and multi-determined parenting. Future studies should further explore the relationship between many diverse influencing factors and the potential mediating or moderating mechanisms among them.

Contrary to our expectations, no relationships were found between infant characteristics and parental role adaptation. Few studies have investigated the association between children’s factors and parental role adaptation, though some previous studies have demonstrated a positive association between birth weight and parental role adaption (Xiao, 2021; Li, 2022). Rajabi et al. (2018) reported that increasing birthweight results in increased adaption to the mother role. However, these relationships were not found in the present study. Future studies should explore the correlations between these factors.

Several limitations existed in this study. First, the study participants were recruited from only two tertiary hospitals in Hubei Province (China) via convenience sampling, so the external validity of the study results may be limited. Thus, further studies that include people from multiple regions should be undertaken. Second, the cross-sectional design of this study constrained the inference of the causality between identified influencing factors and parental role adaptation. Additional studies with longitudinal design are required to verify the causal relationships. Third, other influencing factors that were not examined in this study could also affect the parental role adaptation of parents with preterm infants, such as depression, parenting knowledge, parenting skills, and family function. Therefore, further studies that explore the associations between parental role adaptation and these possible variables could yield more insights.

In conclusion, Chinese parents of preterm infants experience a moderate level of parental role adaptation when their children are discharged from the hospital to home, and the level of parental role adaptation is affected by individual and sociocultural factors. First-time parents, education level, coping and adaptation level, residence location, monthly family income, and perceived social support were all found to be significant influencing factors for parental role adaptation in this study. In view of this, healthcare providers should pay more attention to first-time parents, those with lower education levels, those living in rural areas, and those with lower family incomes. Our results also emphasize that parents should be encouraged to improve their coping and adaptation abilities and to utilize their formal and informal social support networks. Targeted interventions and educational training for parents based on their needs should be formed to promote parental role adaptation and improve the quality of home care for preterm infants, in turn, improving the prognosis and healthy development of preterm infants.

## Data availability statement

The raw data supporting the conclusions of this article will be made available by the authors, without undue reservation.

## Ethics statement

The studies involving humans were approved by Wuhan University Medical Ethics Committee. The studies were conducted in accordance with the local legislation and institutional requirements. The participants provided their written informed consent to participate in this study.

## Author contributions

JL: Project administration, Methodology, Investigation, Conceptualization, Writing – review & editing, Writing – original draft, Software, Data curation. XZ: Supervision, Resources, Data curation, Writing – original draft, Investigation. FY: Validation, Methodology, Data curation, Writing – review & editing, Investigation. XC: Funding acquisition, Supervision, Writing – review & editing, Resources, Conceptualization. LY: Project administration, Supervision, Writing – review & editing, Methodology, Conceptualization.
